# Neural dynamics of prediction and surprise in infants

**DOI:** 10.1038/ncomms9537

**Published:** 2015-10-13

**Authors:** Sid Kouider, Bria Long, Lorna Le Stanc, Sylvain Charron, Anne-Caroline Fievet, Leonardo S. Barbosa, Sofie V. Gelskov

**Affiliations:** 1 Brain and Consciousness group, Laboratoire de Science Cognitive et Psycholinguistique,(EHESS/CNRS/ENS), Ecole Normale Supérieure, PSL Research University, 29 rue d'Ulm, 75005 Paris, France.; 2 Science Division, Department of Psychology, New York University Abu Dhabi, Abu Dhabi, UAE; 3 Vision Sciences Laboratory, Department of Psychology, Harvard University, 33 Kirkland St, Cambridge, Massachussetts 02138, USA

## Abstract

Prior expectations shape neural responses in sensory regions of the brain, consistent with a Bayesian predictive coding account of perception. Yet, it remains unclear whether such a mechanism is already functional during early stages of development. To address this issue, we study how the infant brain responds to prediction violations using a cross-modal cueing paradigm. We record electroencephalographic responses to expected and unexpected visual events preceded by auditory cues in 12-month-old infants. We find an increased response for unexpected events. However, this effect of prediction error is only observed during late processing stages associated with conscious access mechanisms. In contrast, early perceptual components reveal an amplification of neural responses for predicted relative to surprising events, suggesting that selective attention enhances perceptual processing for expected events. Taken together, these results demonstrate that cross-modal statistical regularities are used to generate predictions that differentially influence early and late neural responses in infants.

## Abstract (web summary)

Prior expectations shape neural processing in the brain and violations of these expectations can have a profound influence on learning. Here the authors demonstrate that such predictive coding mechanisms are already functional in the brains of 12-month-old infants.

## Graphical abstract

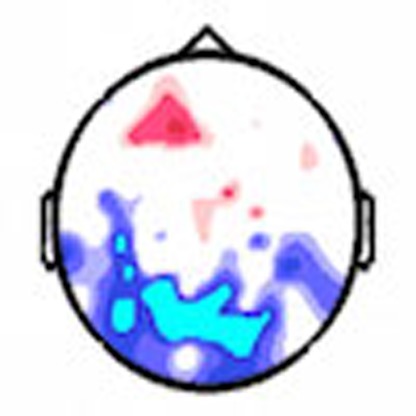

Predictive mechanisms constitute a core aspect of brain function. In order to deal with an ever changing and uncertain environment, perceptual processes do not rely solely on bottom-up responses to sensory inputs. Instead, they also integrate top-down signals that convey predictions and beliefs about upcoming events^1^,^2^,^3^. Under this perspective, perception is thought to involve an inferential process of Bayesian integration, in which prior knowledge, including expectations about upcoming events, is combined with the likelihood information conveyed by the current stimulation. Bayesian accounts have been successfully applied to perceptual decision-making^4^ and sensori-motor learning^5^. These findings are accounted for by predictive coding theories^6^,^7^,^8^, which make the strong assumption that the brain is primarily meant to detect violations of expectations, resulting in an increased propagation of prediction error signals for unexpected events. In this framework, neural systems learn the statistical regularities inherent in the natural world and reduce redundancy by removing the predictable components of the input, transmitting only ‘surprise' (that is, prediction error). Consistent with this view, a recent surge of imaging studies in adult populations has confirmed that prior expectations shape neural responses in sensory regions^9^,^10^. Yet, it remains unclear how these top-down neural mechanisms develop in the infant brain.

Behavioural evidence suggests that infants use statistical regularities to form probabilistic inferences and accordingly adapt their learning strategies across a wide range of domains, including language, spatiotemporal properties or object features (see refs 11, 12 for a review). For example, 8-month-old infants make distinct probabilistic inferences about a sample depending on the population from which it was drawn (for example, they expect a ping-pong ball to be red, when drawn from a population of four red and one white balls)^13^, and look longer when their probabilistic expectations are violated (for example, when a white ball is then drawn instead of a red ball). Similarly, 12-month-old infants can compute estimations of event probabilities by relying on rational expectations consistent with a Bayesian ideal observer^14^. These behavioural studies suggest that infant cognition relies to some degree on mechanisms of Bayesian integration. However, despite a wealth of literature supporting an account of infant cognition as probabilistic inference^11^, it remains largely unknown how neural responses in the infant brain are affected by prior information. In particular, it remains unclear whether and how infants combine prior beliefs with incoming stimulation to build up a coherent representation of their environment. Indeed, while infants might adapt to their environment through passive, bottom-up processes encoding incoming sensory information, they may also rely strongly on their beliefs about the world (that is, predictions) to direct their cognitive resources, constrain their perception, and learn from surprising events (that is, prediction errors).

In the present study, we aimed at addressing this issue in the context of perceptual processing. Specifically, we asked whether and how infants' neural responses to visual events are affected by their prior expectations. We combined high-density electroencephalography (EEG) recordings with a cross-modal cueing paradigm in which auditory cues acted as predictive signals about upcoming visual events (Fig. 1). Twelve-month-old infants (*N*=28) heard two sounds that were predominantly associated with two visual categories (that is, faces versus flowers); the association between these arbitrary sounds and visual categories was counterbalanced across infants. The use of an arbitrary, cross-modal mapping allowed us to ensure that infants', expectations would be driven by top-down mechanisms rather than the processing of local, low-level regularities, or adaptation effects^15^,^16^,^17^ (see Discussion). Furthermore, this design allowed us to address whether prior expectations about upcoming visual categories would impact early or late modulations of EEG components associated with visual responses (that is, in occipito-temporal electrodes). In particular, we were interested in establishing whether, in infants, the top-down impact of prior expectations occurs at early, local, and non-conscious stages of neural activity in sensory cortex, or rather at later processing stages involving large-scale computations, and associated with perceptual consciousness in infants and adults populations^18^,^19^. Our results reveal that the infant brain relies on two complimentary systems for prediction: first, an early attentional amplification of neural activity to visual events that confirm prior expectations, and then, conversely, a late neural amplification to surprising, unexpected events.

## Results

### Cross-modal cueing paradigm

**Figure 1:**
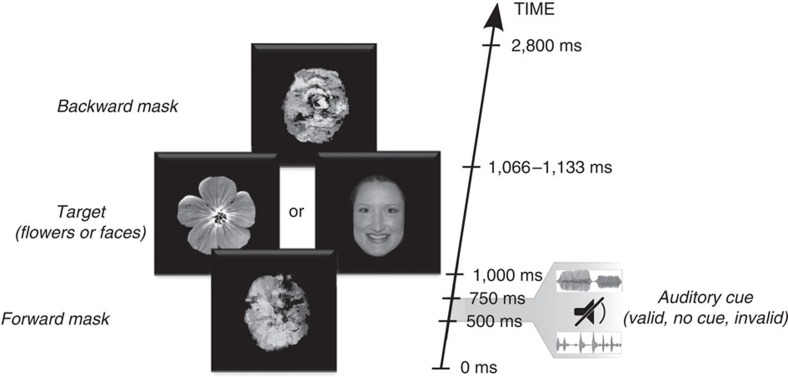
Schematic description of the cross-modal cueing paradigm. Infants were presented on each trial with a visual sequence consisting of a forward mask for 1,000 ms, a critical visual stimuli for 100±33 ms and a backward mask for 1,700 ±33 ms. The critical stimulus was randomly selected from a set of 18 face or 18 flower pictures. In two-thirds of the trials, one out of two sound stimuli (250 ms) was presented 500 ms before the onset of the critical visual stimuli. Each sound stimulus was previously associated with a corresponding visual category (faces, flowers) during a familiarisation phase, by presenting them simultaneously and congruently on each trial. During the experimental phase, the sound stimulus predicted its associated visual category 75% of the time (valid trials), while they preceded the other, unassociated visual category 25% of the time (invalid trials). On the remaining one-third of the trials, no auditory cue was presented (baseline trials).

Infants first learned to associate an auditory cue (sounds ‘A', ‘B') with a corresponding visual category (faces, flowers). During this familiarisation period, visual stimuli were presented simultaneously with their corresponding auditory cue (that is, sound ‘A' always appeared at the same time as a face, sound ‘B' always appeared at the same time as a flower, counterbalanced across participants). Synchronous presentation was preferred to asynchronous presentation during this familiarisation phase because it increases the probability of learning an arbitrary mapping^20^. Then, infants received experimental trials in which the sounds now preceded the visual stimuli by 500 ms on two-thirds of the trials (Fig. 1). On these trials, the auditory sounds predicted their associated visual category 75% of the time (valid trials), while they preceded the other, unassociated visual category 25% of the time (invalid trials). In the remaining one-third of the trials, we used a baseline ‘no-cue' condition in which visual targets were presented without a preceding sound. This condition allowed us to address whether any modulations of sensory signals by predictive mechanisms were specifically driven by expected events (that is, valid trials) or rather by surprising events (that is, invalid trials). This no-cue condition was preferred to an uninformative, third auditory cue (that is, 50/50 probability), as a third cue might confuse infants and reduce their learning abilities. Lastly, in order to maximize the impact of prior expectations on sensory signals^17^, visual stimuli were presented at threshold during experimental trials. This was achieved by using visual masking and a brief stimulus presentation (100±33 ms), approximating infants perceptual threshold at this age^21^.

### EEG components of interest

We first examined which main event-related potential (ERP) components (collapsed across conditions) were induced by the visual targets in this experiment. We focused on a set of occipito-temporal electrodes that were chosen for their sensitivity to face-related components in a similar masking paradigm^18^. This first step allowed us to identify components of interest and their time-windows independently of stimulus category or validity (Supplementary Fig. 1). First, we observed an early positivity corresponding to the P1 that has previously been observed in studies of both face and object perception^22^. The P1 was followed by two components, namely an N290 and a P400, that are classically associated with face processing in infants^22^,^23^. The putative N290 waveform did not directly transfer into a proper negativity (that is, below baseline level), likely because in the current study the stimulus of interest was temporally surrounded by both forward and backwards masks that may have interacted with target processing. Because of its rather small size and proximity to the larger P1, we chose to collapse the putative N290 and the P1 waveforms and consider them as a single ‘P1' component in further analyses. Finally, we observed a sustained and rather late negativity, starting at 700 ms and peaking around 1,000 ms, corresponding to a late slow wave (LSW). This late response has been linked to recognition memory in infants but also more generally to attention and novelty detection^22^,^23^,^24^,^25^. Furthermore, as discussed below, the LSW has been recently shown to constitute a neural marker of perceptual consciousness in infants^18^.

### Cue validity differentially influence early and late EEG responses

**Figure 2:**
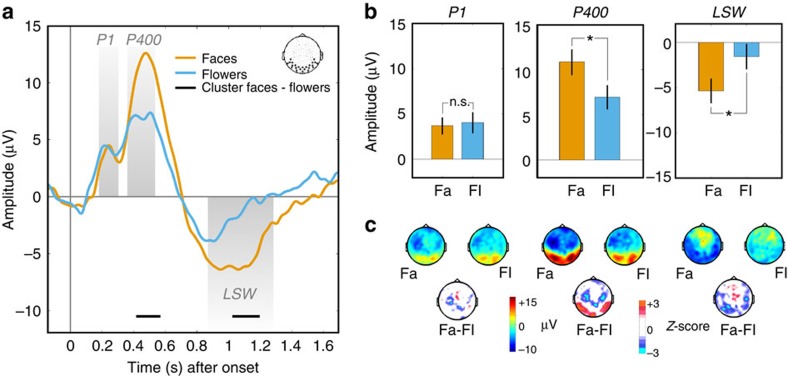
Event-related potentials to faces and flowers. (**a**) Time plots showing the ERPs evoked by faces and flowers (collapsed across the valid, invalid and no-cue conditions) over occipito-temporal electrodes, along with significant clusters of the difference between the two conditions (Monte-Carlo *P* value<0.05). (**b**) Bar plots illustrating voltage differences for the three components of interest (P1, P400 and LSW, see Supplementary Fig. 1). For each component, two-tailed pair-wise comparisons (*n*=28 subjects) were performed on signal amplitudes for face (Fa) and flower (Fl) target stimuli (asterisks denote *P<0.05 and **P<0.01; Error bars show s.e.m.). (**c**) Scalp topographies showing the voltage ERPs evoked by face and flower trials for each temporal window of interest (top row), and their difference in terms of statistical maps (*Z*-scores; bottom row).

We then performed a three-way analysis of variance to inspect how these three components of interests (P1, P400 and LSW) were modulated by target category (faces versus flowers) and by cue validity (valid, invalid). As these components have different (positive or negative) polarities, their modulations are not directly comparable. To circumvent this issue, statistical analysis were performed by inverting the sign of the negative LSW component for a more direct comparison with the other, positive components. This analysis revealed both a main effect of component (F(2,54)=6.08; *P*=0.004), and of target category (F(1,27)=6.60; *P*=0.016), reflecting an increased responses to faces compared with flower stimuli, and an interaction between component and cue validity (F(2,54)=7.23; *P*=0.002). Although the interaction between target category and component did not reach significance (*P*=0.135), *post hoc* comparisons revealed that, consistent with previous literature^22^, the earlier P1 response was insensitive to stimulus category (*P*_*P1*_=0.784; two-tailed; Fig. 2), while the P400 response was larger for faces compared with flowers (*P*_*P400*_=0.023). Interestingly, we also found that the LSW was larger for faces compared with flowers (*P*_*LSW*_=0.040), which extends previous findings showing a stronger LSW for faces compared with non-object patterns^18^.

**Figure 3:**
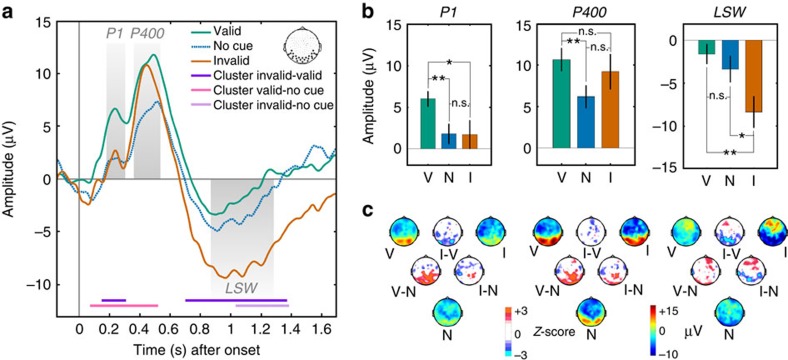
Event-related potentials to valid, no-cue and invalid conditions. (**a**) Time plots showing the ERPs evoked by valid, no-cue and invalid conditions, along with significant clusters of the difference between conditions (Monte-Carlo *P* value<0.05). (**b**) Bar plots illustrating voltage differences for the three components of interest (P1, P400 and LSW). For each component, two-tailed pair-wise comparisons (*n*=28 subjects) were performed on signal amplitudes for valid (V), no-cue (N) and invalid (I) conditions (asterisks denote *P<0.05 and **P<0.01; Error bars show s.e.m.). (**c**) Scalp topographies showing the voltage ERPs evoked by valid, no-cue and invalid trials for each temporal window of interest, and their difference in terms of statistical maps (*Z*-scores).

After verifying that our EEG paradigm was sensitive to category information, we turned to our main manipulation of interest, that is, the impact of a predictive auditory cue on the processing of visual targets. In order to better understand the significant interaction between component and cue validity reported above, we conducted *post hoc* comparisons. Interestingly, except for the intermediate-latency P400 waveform, we found that both early (P1) and late (LSW) components were modulated by prior expectations (Fig. 3). Unexpectedly, however, we found that the auditory cue biased the early and late responses in opposite ways. Indeed, our data revealed that the early component was amplified for valid targets (*P*_*P1*_=0.0144) while the late component (LSW) showed, conversely, a substantial amplification for invalid targets (*P*_LSW_=0.0013). This result suggests that early sensory responses are primarily affected by the validity of predicted events, while late responses may primarily reflect sensitivity to a violation of expectations.

This description was confirmed in further analyses comparing EEG responses for valid and invalid trials relative to the baseline trials (that is, trials without a sound cue). Indeed, for the early component, responses to valid trials were larger relative to responses to both invalid trials and baseline trials (*P*_*P1*_=0.003), while responses to invalid and baseline trials were similar (*P*_*P1*_=0.97). Conversely, for the late component, the valid and baseline conditions remained at the same level, while the response amplification for the invalid condition occurred in comparison to the baseline condition (*P*_LSW_=0.016). This last aspect of the data further underscores our finding that expected and unexpected visual events differentially affect early and late sensory responses.

Interestingly, although there was no modulation by predictive cues for the intermediate component (P400), the mere presence of a preceding auditory stimulus, regardless of its validity, largely increased the amplitude of this component (comparison of cue versus no-cue trials: *P*_P400_=0.005; Fig. 3). One possibility is that the auditory cues also acted as a general boosters of arousal, amplifying the processing of upcoming objects and faces, irrespective of how expected they were. Note that this result permits to rule out the alternative possibility that the no-cue condition conveyed some strong elements of surprise, given the fact that the visual target could suddenly appear without a preceding auditory cue. Although this hypothesis would actually predict a stronger response in the no-cue condition, this was not the case for any of the three components, whether this response was compared with the validly or invalidly cued trials, rendering this interpretation rather unlikely.

Could these effects be driven by responses to only one of the two visual categories (that is, only by trials with faces or flowers)? Further analysis discarded this possibility. Indeed, there was no interaction between target category and cue validity for any of the three components (all *F*s<1), revealing that neural response to both visual categories were similarly modulated by the predictive auditory cues (see Supplementary Fig. 2). A potential issue concerns the fact that the electrodes used in our study might be specially sensitive to face stimuli, but not to the other, flower category, given that they were selected from a previous study^18^ comparing faces versus masking patterns. Yet, although EEG responses were stronger for faces, their spatial distribution was similar across the two categories (Supplementary Fig. 3A). Moreover, the modulation of EEG response by predictions remained present whether we decreased or increased the size of the cluster of electrodes by a third (Supplementary Fig. 3B–D), confirming the robustness of our finding. Lastly, we asked if our results could be biased by the selection of specific temporal windows of interest for our three main components. To address this question, we performed an analysis on the whole time series to identify which temporal clusters showed a significant difference between conditions after performing a non-parametric cluster-based permutation procedure^26^ (see Methods). These cluster-permutation analyses confirmed the results obtained with the windows of interest (compare Figs 2a,b and 3a,b).

## Discussion

Our study shows that cross-modal statistical regularities can bias visual responses in the developing brain, revealing how the neural mechanisms underlying perception are affected by prediction in infancy. After learning the arbitrary mapping between a sound and an object category, infants transformed this meaningless sound into a strong prior that provided predictive information about upcoming visual events. Our finding extends previous behavioural research on the development of probabilistic inferences^11^,^13^,^14^ by revealing how they impact the temporal dynamics of neural responses in infancy. Our approach combining predictive cross-modal cueing with infant EEG recordings revealed that the neural impact of predictive cues follows different dynamics depending on whether visual stimuli were expected or, instead, surprising.

Indeed, while our study confirms the presence of an amplified response for surprising events during late processing stages, we observed, conversely, an amplification of neural responses for the expected events during early processing stages. Although the former result is consistent with predictive coding accounts of perception, the latter result appears to be at odds with this framework. Indeed, from the perspective of predictive coding, the brain is primarily meant to detect violations of expectations, resulting in an increased response corresponding to prediction error signals^6^,^7^,^8^. A straightforward interpretation is that this modulation reflects selective attention, with the auditory signal acting not only as a predictive cue but also an attentional cue amplifying the expected features at the expense of the unexpected ones during the earlier processing stages. Indeed, in Posner paradigms^27^, which are similar to the one used in this study, the impact of a probabilistic cue conflates the effect of attention and the effect of expectations about the location or feature of a forthcoming target^28^. The same P1 component observed in this study is amplified when stimuli are preceded by a spatially valid cue in both adults^29^ and infants^30^. Moreover, this early amplification is exacerbated by the use of challenging perceptual conditions involving barely visible stimuli^31^, such as in the current study. Importantly, although attention and expectations are conflated in most experimental settings, they can be distinguished by their opposite impact on neural responses: while neural activity for attended information is amplified, it is reduced for expected information. Indeed, attention and expectation appear to serve distinct functions: expectation facilitates visual perception by restricting inferences on the basis of prior likelihood, while attention alleviates the systems' computational burden by prioritizing processing of the subset of visual information that appears to be of highest relevance to the organism's goals^28^.

Our results fit within a framework where perception, at least in infants, involves two distinct mechanisms that rely upon prior information: first, an early mechanism of selective attention aimed at amplifying target features that match template information generated by the auditory cue, and then a second mechanisms of Bayesian integration aimed at generating an error signal when the unexpected target appears instead of the expected one. Though, an alternative possibility is that both these effects of attention and expectation are subserved by a unified mechanism of predictive coding. Indeed, recent accounts of predictive coding have proposed that attention is actually subserved by the same mechanisms as those underlying expectation^32^. According to this approach, attention reflects an increase in the precision of prior information, while surprise reflects an increase in the error signal resulting from the mismatch between prior and novel information. Further research should attempt to disentangle how attention and prediction mechanisms interact during development.

Another interesting questions raised by our results concerns whether the late effect of violation of expectations reflects a prediction error signal *per se*, or rather a learning process involving the update of prior beliefs as a function of statistical contingencies^11^. Consistent with this interpretation, a recent behavioural study demonstrated that infants use violations of expectations as specific markers of learning and exploration^33^. When 11-month-old infants were presented with scenes containing objects that violated their expectations (for example, a toy-car hanging mid-air instead of falling when pushed over a ledge) learning was subsequently enhanced and information-seeking behaviours promoted (for example, when infants were handed the toy-car that previously defied gravity, they played with it by dropping it). More importantly, infants in this study learned more effectively about objects that committed violations, and explored them more in order to engage in hypothesis-testing behaviours about the particular kind of violation they encountered. Thus, learning from surprising events could be an important determinant of the late surprise mechanism we observed in the brain of 12-month-old infants.

The observation that prediction error signals involves the LSW component suggests that violations of expectations, at least within the current design, may reflect a conscious stage of perceptual processing. Previous research has shown that perception unfolds in two stages: a preliminary, non-conscious, linear processing stage in which activation is limited to visual regions and reflect the continuous accumulation of sensory input (for example, stimulus duration), and a later, nonlinear stage in which information is passed reciprocally between sensory cortex and higher-order areas, and observed only when the stimulus is consciously reported^19^. In adults, the ERP component that reflects this second stage is the P300 response, as it remains specific to consciously reportable stimuli^34^,^35^,^36^ and involves, additionally, a nonlinear transition as a function of stimulus energy^37^. In infants, a recent study has confirmed the observation of a late, nonlinear electrophysiological component responding only to faces presented above infants' psychophysical threshold, namely the LSW^18^. Because the LSW shares the same characteristics as the P300 in adults, although with a much delayed latency, it has been argued to constitute a neural marker of perceptual consciousness. Previous studies have linked the P300 not only with the identification of conscious stimuli but also with the processing of unexpected events (for reviews see refs 38, 39). Indeed, the P300 is also commonly used in adults not only as a neural signature of conscious access but also as a neural marker of surprise, as it is inversely correlated with the probability of occurrence of a stimulus in odd-ball paradigms^40^,^41^. Thus, we argue that the LSW may reflect a conscious response to violations of expectation in our paradigm. However, whether or not infants require conscious access to learn statistical relationships remains a question for future research. Indeed, it remains possible that infants rely on non-conscious mechanisms of learning, even for the mapping of cross-modal elements, while they express a conscious response to novel or surprising events.

Importantly, the observation of a late rather than an early effect of violation of expectations arguably reflects the cross-modal cueing manipulation used in this study. As mentioned above, we chose a cross-modal, arbitrary mapping to ensure that any effects of prediction were the result of top-down mechanisms rather than sensitivity to local, low-level regularities or adaptation effects. Of course, this is not incompatible with the possibility of earlier and more local forms of prediction error, such as the mismatch negativity (MMN) observed in adult and infant populations^42^,^43^,^44^,^45^. Unlike the P300 observed in adults, the MMN reflects an early, preattentive and nonconscious response to the breaking of low-level regularities within the same sensory modality^16^,^42^,^46^,^47^,^48^. Thus, it remains possible that a manipulation involving cues and targets from the same modality would result in earlier effects of violations of expectations. Such early mismatch effects might be observed not only for low-level sensory information but also for more abstract types of information (for example, infants' responses to numerical violations^49^), as long as it involves within-domain computations (that is, core systems^50^). Note, however, that the large majority of paradigms involving within-domain violations of expectations are explainable in terms of simple adaptation: neurons are first tuned to encode a specific stimulus representation (for example, an orientation, a number) and have a weaker response when the same stimulus reappears compared with a novel stimulus^51^. Interestingly, infants' EEG response to numerical violations (that is, infants see two toys being hidden but then discover a single one^49^) and to numerical adaptation (that is, neural response to same versus deviant number of items across trials^52^) display similar temporal characteristics with early effects around 300–400 ms in both cases, making it plausible that they are sustained by a common mechanism. A notable exception to the confound with adaptation is the observation of an early increase in sensory activity for omissions of expected sound beats in newborn sleeping infants^53^. Here the fact that the neural response increases when the stimulus is actually absent clearly argues for the elaboration of a prediction error signal. Furthermore, the fact that infants were asleep and presumably unconscious corroborates the hypothesis that this low-level prediction error signal results from local and automatic forms of computation. Consistent with this hypothesis, the MMN response to local sound irregularities in adults can be observed during sleep, while high-level prediction error signals associated with conscious novelty detection, as indexed by the P300, are fully abolished during sleep^54^.

In conclusion, we found specific neural responses reflecting early attentional amplification for expected events and, conversely, late surprise effects for unexpected events in 12-month-old infants. These results strongly suggest that the neural mechanisms reflecting the top-down biasing of visual processing by both attention and expectations are functional in infants, but follow distinct temporal dynamics. Of particular interest is the observation that neural responses to surprising events only reflect late components associated with conscious perceptual processes in this cross-modal cueing paradigm. It remains possible, though, that the first stages of neural processing rely on similar mechanisms of probabilistic inference when violations involve local regularities within a domain or sensory modality. For more abstract, cross-modal predictions about the nature of upcoming events, infant might have to engage in effortful learning strategies involving global broadcasting at the neural level^19^, and thus requiring conscious inferences. Consistent with this hypothesis, the recent finding that infants learn better from unexpected events^33^ might derive from conscious processes linked to working-memory maintenance and hypothesis testing rather than within-domain forms of learning and computations. Future studies should focus on the neural basis of simple versus complex forms of probabilistic inferences, and their impact on learning. Whether infants learn unconsciously or require consciousness for more complex forms of learning remains a fundamental question for future research.

## Methods

### Participants

A total of 28 infants participated in this study (14 girls; mean age=12 months 5days; age range=(11 months 23 days to 12 months 18 days); s.d.=7 days). An additional two infants participated in the study but were excluded from analysis due to too few artefact-free trials (that is, not having at least two trials in each of the six conditions of interest). All infants were born at term. The study was approved by the regional ethical committee for biomedical research. Parents gave their written informed consent before starting the experiment.

### Stimuli

Visual stimuli consisted of greyscale pictures of 18 faces, 18 flowers and 30 masking patterns matched for overall luminance, contrast and size. Faces consisted of smiling, female faces. Flowers were chosen as an object that had the same overall elliptical shape as a face but none of its characteristic features, so that both faces and flowers could be masked by the same patterns. Masking patterns were constructed by overlaying images of faces and other elliptical stimuli (watches, flowers and so on), turning them upside-down and finally scrambling the layers to ensure the absence of visible face or object features. Auditory stimuli were two perceptually distinctive sounds constructed by modifying pre-existing sounds: a recording of a toy and a sound present in the Mac OX sound library. Both sounds were adjusted to have an equal length of 250 ms and a transition between pitches at approximately the same moment (∼135 ms after onset) in the middle of the sound. They also were equalized to have identical root-mean squares and mean amplitude.

### Experimental procedure

Infants sat in the lap of their parent, eyes at the level of the stimuli and about 55 cm from a 21″ CRT screen (at 60 Hz) while wearing a high-density EEG-net. Parents wore both opaque sunglasses and noise-cancelling headphones with masking music to ensure that they could not influence their infant's response. Two hidden loudspeakers were placed behind the screen. A hidden camera located underneath the screen allowed the experimenter, who could see the infant but not the stimuli, to register online for each trial whether the infant was looking or not towards the screen. This coding was then used in later analysis to reject trials when infants were not watching. Stimuli were presented using the Psychophysics toolbox for Matlab^55^. During the familiarization phase, face and flower stimuli chosen randomly from the stimulus list were presented in alternation for 1 s each, with a 1.5 s delay and for a total of 10 trials. Each visual stimulus was presented simultaneously with a given auditory cue (that is, both onsets were synchronous). This simultaneous presentation procedure was aimed at increasing the likelihood of associating each auditory cue with the corresponding visual stimulus category. During the test phase, infants received blocks of nine trials followed by a feedback event. During a trial, infants saw a forward mask, eventually an auditory cue (that is, a sound associated with faces, a sound associated with flowers, or no sound), then a visual target stimulus (that is, a face or flower stimulus), and finally a backward mask. The forward mask was chosen randomly and presented for 1,000 ms. When an auditory cue was presented, it started 500 ms after the onset of the forward mask, and it was played for 250 ms. Then, immediately following the forward mask, the visual target appeared on the screen for either 66, 100 or 133 ms. These durations were chosen because they approximate the psychophysical thresholds of infants face perception^21^. These durations were collapsed in further analysis in order to increase the signal-to-noise ratio, mainly due to the restricted number of trials in the invalid condition (that is, 25% of the cued trials; 16.7% in total, when including the no-cue trials). Target stimuli were followed immediately by a backward mask, which lasted 1,800 s minus the duration of the target stimuli. At the end of each block, infants received a feedback event in which either a face or a flower stimulus, selected randomly, was fully visible (that is, presented for 3 s) and surrounded by coloured crosses, simultaneously with its corresponding predictive sound repeated every second. This feedback event, thus, appeared after every nine test trials and was inserted to keep infants attentive to the presentation display. An inter-block interval of 1,500 ms was used, in which the screen only contained a single large white empty circle on the black background.

### Design

The design of this experiment involved 1/3 of trials with no sound preceding the visual target (no-cue condition) and 2/3 of trials with a predictive cue. In the latter case, each of the two auditory cues predicted with 75% accuracy the presence of either faces or flowers (valid condition), while the visual target did not match the auditory cue in 25% of the sound trials (invalid condition). The combination of sound type, target stimulus type, and target duration was chosen semi-randomly from a matrix of all conditions with the pre-determined probabilities that in each block of nine trials, each target duration was presented equally (three presentations of each of the three durations per block) to prevent the possibility that a block would contain mostly sub-threshold trials. Which of the two physical sounds predicted the visual target category was counterbalanced across participants (for example, sound ‘A' predicted face-stimuli for one half of participants, while the same sound predicted flower stimuli for the other half).

### EEG recordings and data processing

The electroencephalogram was continuously digitized at 250 Hz using a 128-electrodes HydroCel net (EGI; Eugene, OR, USA) referenced to the vertex. The signal was first re-referenced to the average activity, then high-pass filtered at 0.2 Hz, low-pass filtered at 20 Hz and finally segmented from −150 ms to +1,700 ms relative to the onset of the target stimulus. Infant's gaze was constantly monitored throughout the experiment, and any trial in which the infant looked away from the screen was rejected. An average of 22.2 trials (s.d.=10.6) were rejected with this method. For each epoch, channels contaminated by important eye or motion artifacts (that is voltage exceeding±400 μV, or a local deviation higher than 400 μV over a 10 samples window) were rejected and their voltages automatically interpolated using a standard procedure of linear interpolation from the nearest electrodes. Trials with more than 35% contaminated channels were rejected. Furthermore, we applied an artefact correction method recently developed for infants^56^,^57^ which consisted in a temporal smoothing of voltages exceeding±120 μV. The main advantage of this method is that it allows preserving epochs even if they contain aberrant local deviations, by relying on a smoothing matrix minimising the high-amplitude information without distorting other sources contained in the signal. Finally, averages were baseline corrected on the 150 ms period preceding the target stimulus onset. The mean number of artefact-free epochs was 63.6 trials per infant. These trials were averaged per condition, that is target category (faces versus flowers) X cue type (valid, invalid, no-cue), resulting in six conditions of interest. For each condition, the mean number of artefact-free epochs was as follows: face-valid: 15.82 (s.d.=8.04); face-no-cue: 10.43 (5.62); face-invalid: 5.43 (2.61); flower-valid: 16.04 (7.61); flower-no-cue: 10.39 (5.81); and flower-invalid: 5.5 (2.53).

In this study, we used a region of interest approach by focusing on a cluster of occipito-temporal electrodes shown to be particularly sensitive to face-related components in a similar masking paradigm^18^ and corresponding, according to the 10–20 standard positioning system, to electrodes TP9, P9, PO7, O1, OZ, O2, PO8, P10 and TP10. In order to identify the main ERP components evoked by target stimuli independently of the conditions of interest, we first collapsed all trials across conditions and observed three temporal windows of interest: 180–300 ms for the early component, 360–540 ms for the P400 and 860–1,280 ms for the LSW. We then inspected which contrasts were significant for the effects of target categories and cue validity (Figs 2b and 3b, respectively). In addition, statistical significance was assessed through cluster/permutation statistics calculated across participants, allowing us to deal with the potential issue of multiple comparisons in a principled manner (Figs 2a and 3a). Each cluster was constituted by the samples that consecutively passed a specified threshold (in this case sample a *P* value of 0.05). As demonstrated by Maris and Oostenveld^26^, this threshold doesn't change the type-1 error, and the method controls for false alarms independently of this value. The cluster statistics was chosen as the sum of the t-values of all the samples in the cluster. Then, we compared the cluster statistics of each cluster with the maximum cluster statistics computed after a random permutation of conditions within participants, increasing a counter N every time the random cluster statistics was bigger than the real cluster statistics, and repeated this processes 3,000 times. The significance of each cluster was finally assessed by using a threshold Monte-Carlo *P* value N/3,000<0.05.

## Additional information

**How to cite this article:** Kouider, S. *et al*. Neural dynamics of prediction and surprise in infants. *Nat. Commun.* 6:8537 doi: 10.1038/ncomms9537 (2015).

## Supplementary Material

Supplementary Information Supplementary Figures 1-3
