# Supplementary material for: Neural dynamics of prediction and surprise in infants
Source: Nat Commun. 2015 Oct 13;6:8537. doi: 10.1038/ncomms9537 (PMC4633815; doi:10.1038/ncomms9537)
Supplement: Supplementary Information — Supplementary Figures 1-3 [file ncomms9537-s1.pdf]

SUPLEMENTARY FIGURES

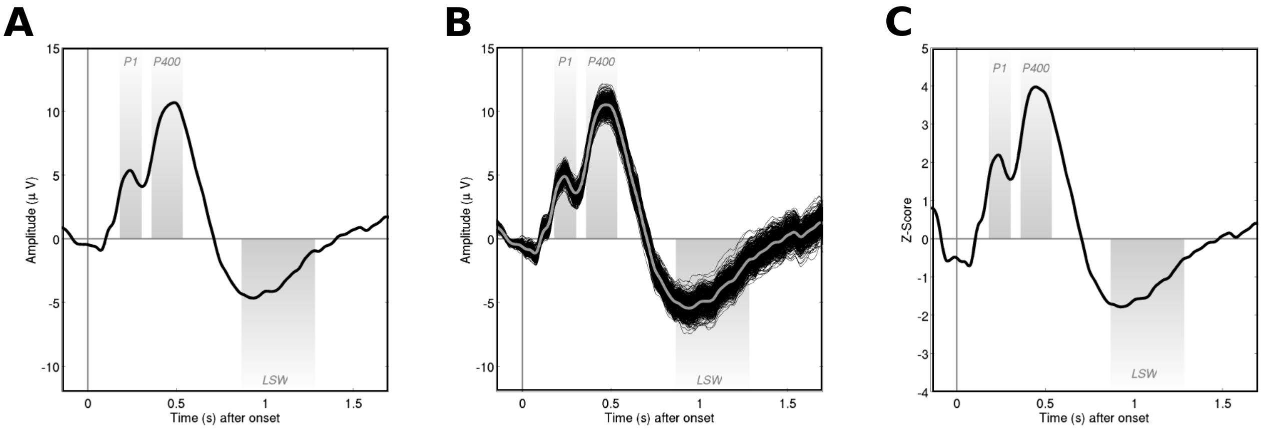

Supplementary fig. 1. **Global ERP waveform over occipito-temporal electrodes.** The global ERP evoked by visual targets revealed three main components of interest: an early P1 component, an intermediate P400 component, and a Late Slow Wave component. Figure S1A shows the main ERP induced by the visual targets collapsed across all conditions and used for delimitating the 3 components of interest used for further analysis. To ensure that our selection of windows of interest was not biased due an unbalanced number of epochs per condition (e.g., more valid than invalid trials), leading to a problem of non-independence between the components of interest and their modulation by the conditions manipulated in this study, two additional analyses were carried out. First, we used a downsampling procedure to take into account the unbalanced number of trials across conditions in our dataset. This procedure consisted in randomly selecting a subset of trials in the valid and no-cue condition, which has the same number of trials as the less numerous invalid condition. This step allowed us to compute a non-biased ERP by averaging across the same number of trials per condition. Using a bootstrap procedure, we repeated this operation 500 times. Figure S1B shows the superimposed ERPs (in black) along with the bootstrap average (in gray). As can be seen in this Figure the 3 main temporal windows for the 3 components of

interest (P1, P400 and LSW) are virtually identical to the ones computed in the first place (compare Figure S1A with S1B). Secondly, we also normalized each condition by dividing the mean by the variance (i.e., z-score) at each sample, and then averaged the 3 waveforms to obtain another unbiased estimate of the condition-independent ERPs (Figure S1C). Here again, we observed the same results (compare Figure S1A with S1C), ruling out the issue of non-independence.

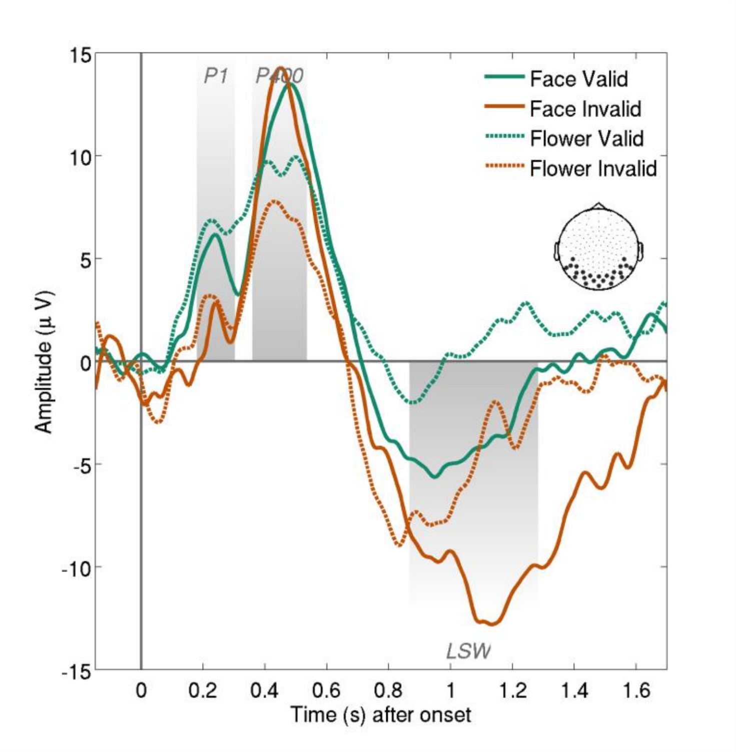

Supplementary fig. 2. Event-related potentials to Valid and Invalid conditions as a function of Target Category.

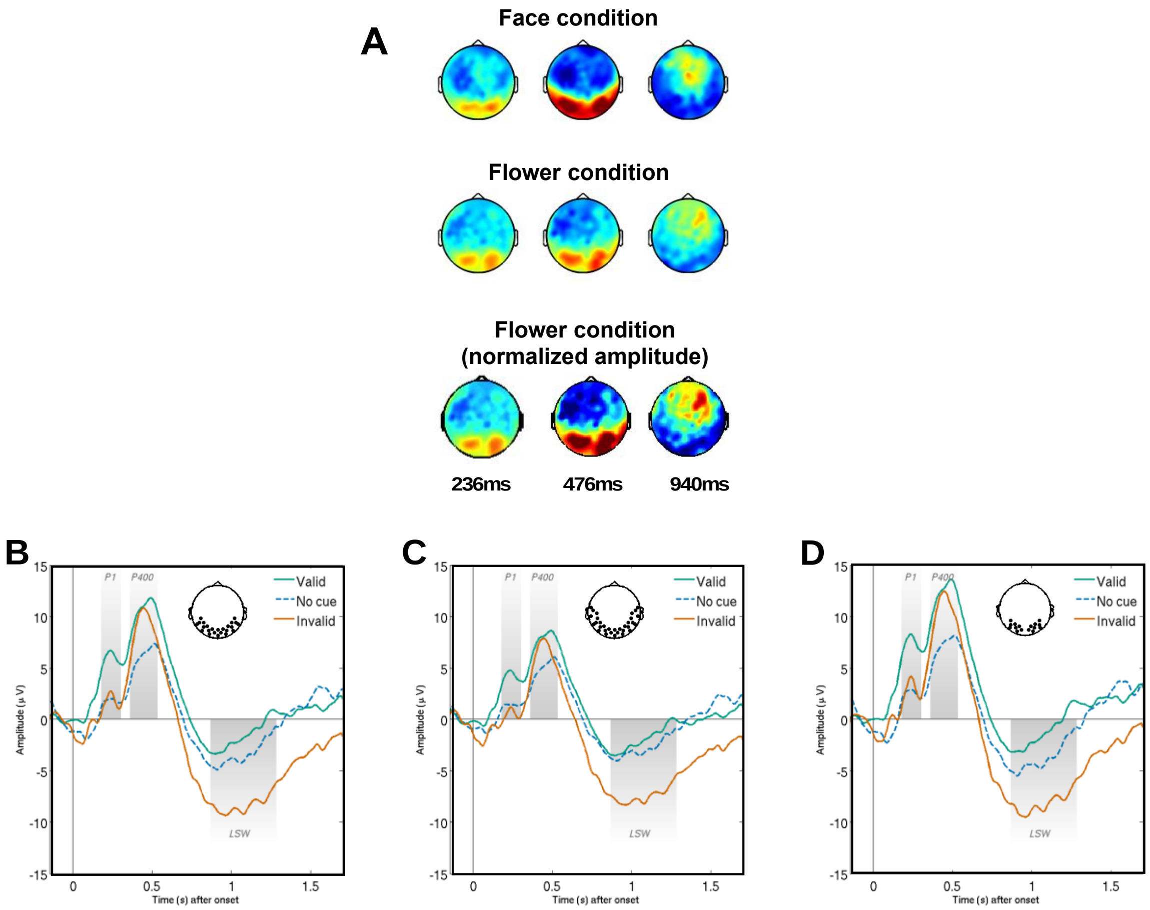

Supplementary fig. 3. Comparison of topographies across target categories, and effects of validity as a function of cluster spatial extent. A) The topographies taken at peaks of the P1, P400 and LSW components show that although the amplitude of EEG responses is stronger for faces compared to flowers, their spatial localization is very similar. Topographies for the flower conditions in the bottom line confirm that the spatial localization remains almost identical across categories especially when normalizing the amplitude across categories (by multiplying the response amplitude for each components by the face/flower amplitude ratio). B-D) We also inspected whether the effects of prediction were specific to the cluster of electrodes we used, by either reducing the occipito-temporal cluster from the 24 original electrodes out of 128 to 16 more occipital electrodes or by extending it more temporally to 32 electrodes. Although the curves are not identical, the effects of validity appear stable across the three cluster extents.
